# The Impact of Gastrectomy on Inflammatory Bowel Disease Risk in Gastric Cancer Patients: A Critical Analysis

**DOI:** 10.3390/curroncol31100430

**Published:** 2024-09-25

**Authors:** Grigorios Christodoulidis, Konstantinos-Eleftherios Koumarelas, Kyriaki Tsagkidou, Eirini-Sara Agko, Dimitra Bartzi, Konstantinos Koumarelas, Dimitrios Zacharoulis

**Affiliations:** 1 Department of General Surgery, University Hospital of Larissa, University of Thessaly, Biopolis Campus, 41110 Larissa, Greece; zachadim@yahoo.com; 2 Department of Emergency Medicine, General Hospital of Larissa, 41221 Larissa, Greece; kostaskoumarelas@gmail.com; 3 Department of Gastroenterology, General Hospital of Larissa, 41221 Larissa, Greece; tsagkidoukyriaki@gmail.com; 4 Department of ICU, Asklepios Paulinen Clinic Wiesbaden, Geisenheimer Str. 10, 65197 Wiesbaden, Germany; agosara44@gmail.com; 5 Department of Oncology, 251 Air Force General Hospital, 11525 Athens, Greece; demmy345@hotmail.com; 6 Department of General Surgery, General Hospital of Nafplio, 21100 Nafplio, Greece; kostaskoum.rsr@hotmail.com

**Keywords:** gastric cancer, gastrectomy, inflammatory bowel disease, microbiome, ulcerative colitis, Crohn’s disease

## Abstract

Gastrectomy, a prevalent surgical procedure for gastric cancer, results in substantial alterations to the gastrointestinal tract, including reduced gastric acid production and significant modifications to the gut microbiota. These changes can impair postoperative recovery, influence metabolic functions, and predispose patients to inflammatory bowel disease (IBD). Studies have shown an increased risk of IBD, particularly Crohn’s disease (CD) and ulcerative colitis (UC), in patients following gastrectomy and bariatric surgeries such as Roux-en-Y gastric bypass (RYGB) and sleeve gastrectomy (SG). For instance, patients undergoing RYGB have a higher hazard ratio for developing CD, while SG patients show an increased risk for UC. The surgical alteration of the gastrointestinal tract promotes dysbiosis, with a significant increase in pathogenic bacteria and a decrease in beneficial microbial populations. This dysbiosis can impair the intestinal mucosal barrier and promote systemic inflammation. Understanding the mechanisms behind these changes and their clinical implications is essential for developing effective postoperative management strategies. Probiotics and enhanced recovery after surgery (ERAS) protocols have shown promise in mitigating these adverse effects, improving gut microbiota balance, and enhancing patient outcomes. Further research is necessary to fully elucidate the long-term impacts of gastrectomy on gastrointestinal health and to refine therapeutic approaches for postoperative care.

## 1. Introduction

Gastric cancer (GC) poses a significant global health concern, responsible for over a million new cases and more than 783,000 deaths annually, being the fifth most common cancer and the third leading cause of cancer-related deaths worldwide [1,2]. Radical gastrectomy, which includes partial or total removal of the stomach, remains the main curative treatment for GC, often supplemented with adjuvant therapies such as chemoradiotherapy, targeted therapy, and immunotherapy [1,3]. Despite its effectiveness, gastrectomy causes major anatomical and physiological changes in the gastrointestinal (GI) tract, leading to numerous postoperative complications and persistent GI symptoms, which can significantly affect a patient’s quality of life [3,4].

The surgical changes in the GI tract, including alterations in pH, oxygenation levels, and biliary diversion, profoundly impact the gut microbiome [3,4]. The gut microbiota, a complex and diverse community of microorganisms, is crucial for maintaining homeostasis, regulating immune responses, and influencing nutrient absorption and metabolism [1,5]. Post-gastrectomy, the gut microbiome undergoes significant dysbiosis, characterized by an increase in typical oral cavity bacteria and potential pathogens, and a decrease in beneficial bacteria [4]. This dysbiosis is associated with various postoperative complications, including intestinal inflammation and impaired recovery [5,6].

Inflammatory bowel disease (IBD), which includes Crohn’s disease (CD) and ulcerative colitis (UC), is a chronic inflammatory condition of the GI tract. Its pathogenesis involves a defective mucosal barrier that allows intestinal bacteria to induce an immune response, leading to chronic inflammation [7]. The incidence of IBD is rising, particularly in regions with higher socioeconomic status, which has been identified as an independent risk factor [7]. In obesity, chronic inflammation results from pro-inflammatory cytokines and adipokines secreted by adipocytes, macrophages, and lymphocytes infiltrating the mesenteric fat, mechanisms that are overexpressed in active IBD [7].

Recent studies suggest an association between bariatric surgery (BS) and the development of de novo IBD, underscoring the need to understand the impact of surgical alterations on gut microbiota and its role in IBD pathogenesis [2,8]. Although BS primarily affects the superior intestine, it also significantly influences distal intestinal physiology and the composition of the gut microbiota, potentially impacting metabolic outcomes and the development of IBD [7,9]. Different BS procedures, such as Roux-en-Y gastric bypass and sleeve gastrectomy, differently alter the gut microbiota, which could influence weight loss, maintenance, and the resolution of related comorbidities [9]. Roux-en-Y gastric bypass has similarities with Roux-en-Y anastomosis after gastrectomy.

Whole genome sequencing and 16S rRNA gene sequencing are some of the novel sequencing techniques which have revolutionized microbial investigation [5]. These advancements enable the identification of previously uncultured bacteria and provide insights into the complex interactions between gut microbiota and host physiology.

Given the increasing incidence of both GC and IBD and the significant impact of gastrectomy on the gut microbiome, it is crucial to explore the link between post-gastrectomy dysbiosis and the development of IBD. This review aims to investigate the incidence and clinical outcomes of IBD in patients undergoing gastrectomy for GC, providing insights into the role of gut microbiota in this complex interplay. Understanding these interactions may offer new perspectives on postoperative management and potential therapeutic strategies to improve patient outcomes [3,5,8,10].

## 2. Materials and Methods

PubMed, Cochrane Library, Medline, Scopus, clinical trial register, and Web of Science databases were initially searched by the authors to retrieve studies reporting data on inflammatory bowel disease and gastrectomy from 2015 to the present day. The following medical subject heading [MeSH] terms alone or matched by the logical operators “OR” or “AND” were used: “Inflammatory Bowel Disease”, “IBD”, “Gastric cancer”, “Gastrectomy”, “Crohn’s disease”, “Ulcerative colitis”, and “Gut microbiota”. Old, repetitive, and non-English studies were excluded. The inclusion criteria were studies related to gastrectomy for either GC or bariatric surgery, which described the postoperative course of patients who either already had IBD or acquired IBD after surgery. After an initial title screening, each relevant article was subsequently reviewed, and 30 representative scientific papers were finally selected.

## 3. Results

### 3.1. The Impacts of Gastrectomy

Gastrectomy, a common surgical intervention for gastric cancer, entails substantial alterations to the gastrointestinal tract. These changes, including reduced gastric acid production and significant modifications to the gut microbiota, have implications for postoperative recovery and overall patient health. Recent studies suggest that these alterations may also predispose patients to inflammatory bowel disease (IBD), adding another layer of complexity to the postoperative management of these individuals.

Moreover, inflammatory bowel disease (IBD), including Crohn’s disease (CD) and ulcerative colitis (UC), has been increasingly observed in patients following gastrectomy for bariatric surgery, as well as gastric cancer.

A significant body of research suggests a heightened risk of developing IBD after bariatric surgeries such as Roux-en-Y gastric bypass (RYGB) and sleeve gastrectomy (SG). Among 64,188 individuals who underwent RYGB, the hazard ratio (HR) for developing CD was 1.8 (95% CI 1.5–2.2), and for unclassified IBD, it was 2.7 (95% CI 2.0–3.7). Conversely, SG patients had an increased risk of UC (HR 1.8, 95% CI 1.1–3.1) but not CD (HR 0.8, 95% CI 0.3–2.1) [11].

In another study, 80 patients developed de novo IBD post-bariatric surgery: 75% were diagnosed with CD and 21% with UC. RYGB was the most common procedure (80%) linked to these outcomes [8]. RYGB can be easily compared to any type of partial/subtotal gastrectomy with Roux and Y anastomosis.

Gastrectomy, which involves reconstructing the gastrointestinal tract and reducing gastric acid, drastically alters the gut microbiota. For instance, subtotal gastrectomy significantly changes the diversity, community composition, and predicted gene functions of gastric microbiota [12]. These alterations can impair the intestinal mucosal immune barrier, leading to systemic inflammatory responses and hindering postoperative recovery [6]. Gastrectomy leads to notable changes in the gut microbiome due to the loss of the gastric barrier. Under normal conditions, the stomach’s acidic environment, with a pH around 2.0, acts as a barrier to pathogenic microorganisms. However, after subtotal gastrectomy, the gastric pH increases to over 6.0 [5,6,7,8,9,13]. This change allows oral cavity bacteria, such as *Streptococcus*, *Veillonella*, *Prevotella*, *Oribacterium*, and *Mogibacterium*, to survive gastric passage and colonize the distal GI tract. Studies have shown that these oral bacteria increase significantly in the gut microbiome of gastrectomized patients, with *Streptococcus* and *Veillonella* being 2.5 to 4 times more abundant compared to controls [6,13,14].

Additionally, the postoperative gut experiences increased oxygen levels, creating a favorable environment for aerobic and facultative anaerobic bacteria. Research indicates that, post-gastrectomy, the abundance of aerobes such as Streptococcus and facultative anaerobes like *Escherichia* and *Enterococcus* increases by up to 50% [13,14,15]. Furthermore, biliary diversion resulting from gastrointestinal reconstruction alters bile acid flow, stimulating the growth of bile acid-transforming bacteria. 

Lin et al. confirmed differences in gut microbial composition between gastrectomy patients and healthy controls [16]. Their study analyzed fecal samples from 28 individuals who had partial gastrectomy for gastric cancer—14 with Billroth II anastomosis and 14 with Roux-en-Y gastrojejunostomy—and 14 healthy controls. The findings showed that both the Chao1 index, which measures bacterial richness, and the Shannon index, which considers both richness and evenness, were significantly higher in the gastrectomy group. This indicates greater microbial diversity among these patients. Specifically, the analysis identified a greater abundance of genera such as *Oscillospira*, *Prevotella*, *Coprococcus*, *Veillonella*, *Clostridium*, *Desulfovibrio*, *Anaerosinus*, *Slackia*, *Oxalobacter*, *Victivallis*, *Butyrivibrio*, *Sporobacter*, and *Campylobacter* in the gastrectomy group. Since some patients had undergone surgery around eight years prior, this suggests that microbial differences may persist long-term [10,17]. 

Horvath et al. further explored this by examining the fecal microbiomes of 14 patients who had upper partial gastrectomy with Billroth II anastomosis and comparing them with eight control relatives. This study found a higher presence of oral bacteria such as *Veillonella*, *Oribacterium*, and *Mogibacterium*, along with *Escherichia*–*Shigella*, *Enterococcus*, and *Streptococcus* in the gastrectomy group. This supports the idea that changes in the oral microbiome due to gastrectomy can influence gut microbial composition. However, the Shannon index was lower in the gastrectomy group compared to controls, a result that may be affected by the small sample size and requires further research [4,10].

Additionally, Liang et al. investigated changes in gut microbiome before and after gastrectomy by comparing fecal samples collected within one week before surgery with those taken post-surgery from six patients who had distal gastrectomy (one with Billroth II anastomosis and five with Roux-en-Y gastrojejunostomy). Their results showed no significant changes in microbial diversity immediately after surgery, suggesting that variations observed in other studies may develop over a longer period [10,18].

The increase in specific taxa such as *Fusobacterium nucleatum*, *Veillonella*, and *Streptococcus* is directly associated with inflammation [4,19]. This dysbiosis is linked to higher incidences of CD and UC post-surgery [20].

The metabolic effects of bariatric surgery (BS), such as Roux-en-Y gastric bypass (RYGB) and sleeve gastrectomy (SG), have been studied extensively. RYGB patients exhibit a better lipid metabolism response with significant decreases in cholesterol levels and blood pressure, while SG patients show improved liver function outcomes [9]. The increase in circulating bile acids and the resultant changes in intestinal microbiota through receptors like the farnesoid X receptor and Takeda G protein-coupled receptor 5 (TGR5) are believed to drive these metabolic improvements. Bile acids, essential in cholesterol metabolism and lipid digestion, also engage in crucial interactions with intestinal microbiota, influencing overall gut health [20,21]. Lymphoid follicles are commonly found in the mucosa of the colon and intestines, especially in children and young animals. As individuals age, these follicles tend to diminish, eventually becoming restricted to Peyer’s patches and the appendix in the distal ileum, as well as the rectum in older adults. Kagiya et al. observed in their animal model that gastrectomy in rats, used to model nodular lymphoid hyperplasia (NLH) in colorectal diseases, showed significantly larger lymphoid follicles post-gastrectomy compared to sham surgery, indicating increased lymphocytic intestinal immunity and alterations in the intestinal environment [19]. Nodular lymphoid hyperplasia (NLH) refers to the nodular proliferation of lymphoid tissue within the intestinal and colonic mucosa, a common feature in the distal ileum. This condition is frequently seen in individuals with infections or idiopathic inflammatory bowel disease, requiring careful differentiation from other pathologies [19].

### 3.2. Risk of Inflammatory Bowel Disease

Emerging evidence suggests that gastrectomy for gastric cancer as well as BS, particularly RYGB, may increase the risk of developing Crohn’s disease (CD) and unclassified IBD. Studies have reported an increased incidence of de novo IBD in patients post-BS, with RYGB showing a higher risk for CD compared to SG, which is more associated with ulcerative colitis (UC) [7,11]. The anatomical changes and resultant dysbiosis following gastrectomy might trigger chronic intestinal inflammation in genetically predisposed individuals [7,8]. Gastrointestinal malignancies are linked to chronic systemic inflammation. Cancer cells produce a range of pro-inflammatory cytokines, including tumor necrosis factor-alpha (TNF-α), interleukin-6 (IL-6), and interleukin-1 (IL-1). These cytokines not only promote tumor growth but also exacerbate gut inflammation, worsening IBD symptoms and driving disease progression. In addition, cancer treatments like chemotherapy and radiation therapy can further damage the gut barrier, increasing intestinal permeability and inflammation. Obesity is also directly linked with inflammation, with adipose tissue serving as an active source of inflammatory mediators. Adipokines, such as leptin and resistin, along with cytokines like TNF-α and IL-6, contribute to systemic inflammation and increase gut permeability. This vulnerability amplifies the inflammatory response, contributing to IBD progression [22,23].

### 3.3. Mechanisms and Implications

The pathogenic mechanisms proposed for IBD development post-gastrectomy include the loss of gastric defenses, increased bile acid levels, rapid weight loss, and changes in gut microbiota composition. For instance, the lower expression of the bile acid receptor TGR5, which protects against colitis, may contribute to increased IBD risk post-surgery [8,21]. Additionally, the increased intestinal transport and survival of bacteria due to reduced gastric acidity could aggravate inflammation and alter microbial communities [4,12].

The development of inflammatory bowel disease (IBD) post-gastrectomy is thought to be driven by several mechanisms. One significant factor is microbial dysbiosis. The surgical alteration of the gastrointestinal tract changes the gut environment, leading to an imbalance in microbial communities. This dysbiosis promotes the growth of pro-inflammatory bacteria that can trigger IBD. Post-gastrectomy, the diversity and composition of gut microbiota are significantly altered, resulting in a reduction in beneficial bacteria and an increase in harmful bacteria. These changes disrupt the delicate balance of the gut ecosystem, promoting inflammation and contributing to the development of IBD.

Another contributing factor is immune dysregulation. Changes in the gut microbiota and bile acids influence the immune system, potentially leading to dysregulated immune responses. The immune system relies heavily on signals from gut microbiota to maintain homeostasis. When this communication is disrupted due to microbial dysbiosis, the immune system can become hyperactive, leading to chronic inflammation characteristic of IBD. Altered bile acid profiles post-gastrectomy can exacerbate this immune dysregulation, further increasing the risk of IBD. Tumor cells can also suppress local and systemic immune responses, weakening the gut’s ability to control inflammation. Immunotherapy treatments can also cause immune overactivation, leading to immune-mediated inflammation in the gastrointestinal tract, which can trigger or exacerbate IBD. Obesity is associated with immune system dysfunction, particularly in macrophages and T cells, which contribute to a heightened inflammatory response in the gut. Obese individuals have elevated levels of pro-inflammatory immune cells, which can exacerbate the immune dysregulation seen in IBD. Malnutrition leads to impaired immune responses, particularly in T cells and macrophages. The reduced function of these immune cells in the gut allows for the persistence of inflammation and infection, worsening the inflammatory response in IBD [22,23].

Nutritional deficiencies following gastrectomy also play a crucial role. Gastrectomy can lead to malabsorption and nutritional deficiencies, which may impair the immune system and mucosal barrier, increasing susceptibility to IBD. Malnourishment can also be caused by GC and obesity. This weakened immune state promotes gut barrier dysfunction, increasing the risk of developing IBD or exacerbating its symptoms. Additionally, micronutrient deficiencies common in malnourished individuals, such as zinc and vitamin D deficiencies, further impair immune defenses in the gut, making the intestinal lining more vulnerable to inflammation [22]. 

Genetic predisposition is another important factor. Some patients may have a genetic predisposition to IBD, which, when combined with the environmental and physiological changes post-gastrectomy, may trigger disease onset. Genetic factors can influence how the body responds to changes in gut microbiota and immune regulation. Individuals with a family history of IBD or specific genetic markers may be more susceptible to developing the disease following the significant physiological changes induced by gastrectomy [23,24].

Gastric cancer cells, as well as Tumor associated fibroblasts excrete exosomes, proteins and RNA molecules responsible for the metastatic dynamic, inflammation and infil-tration. However, these molecules are also associated with gut inflammation. miR-29 (micro-RNA 29) is crucial for immune regulation, affecting interleukin-23 levels in dendritic cells. miR-223 modulates intestinal dendritic cells and macrophages, reducing inflammation in IBD. miR-146b controls macrophage polarization and inflammation, while miR-150 regulates immune cell development and intestinal barri-er integrity. miR-155 enhances T-cell responses and Natural Killer (NK) cell function, contributing to inflammation in IBD. miR-24 influences T cell development and apoptosis, impacting ulcerative colitis. miR-29b-1-5p affects gastric cancer progression and intestinal epithelial cell apoptosis. miR-30c regulates autophagy and Th17 cell differentiation, and it also affects macrophage behavior under hypoxic conditions. miR-106b is linked to disease severity in CD and UC, affecting cell invasion and in-flammation. miR-141-3p prevents normal fibroblast transformation into can-cer-associated fibroblasts and plays a role in immune responses. miR-199a-5p pro-motes cancer progression and contributes to inflammation and stress in IBD (Table 1) [23,24].

Laparoscopic gastrectomy requires the use of CO_2_. Alterations in the peritoneal fluid and microcirculation caused by CO_2_ insufflation during surgery can lead to a more virulent phenotype of gut microbiota, aggravating intestinal inflammation and prolonging postoperative recovery. Huang et al. observed that *Lactobacillus* and *Bifidobacterium*, which are beneficial bacteria, were more abundant in the deep Neuromascular Block (NMB) group, while the moderate NMB group had higher levels of *Dialister*, which is associated with constipation [25]. Additionally, the deep NMB group exhibited higher levels of *Desulfovibrio*, which negatively correlated with intestinal inflammation.

An increase in oral and aerotolerant bacteria, coupled with alterations in bile acid composition, is associated with intestinal inflammation, small intestinal bacterial overgrowth (SIBO), and a higher risk of colorectal cancer [26]. Erawijantari et al.‘s metabolome and microbiome analysis found elevated levels of deoxycholic acid, a secondary bile acid, in patients who underwent gastrectomy. This increase is thought to result from changes in bile flow following the surgery, which promotes the growth of bacteria that transform bile acids. Studies show that bile acid malabsorption, often seen with extensive resections of the terminal ileum, exacerbates IBD symptoms such as bile acid-mediated diarrhea and impaired fat digestion [12,16]. For example, in patients with resection of the terminal ileum, bile acid-mediated diarrhea occurs in approximately 40% of cases [16]. Recent research also suggests that certain bile acids may influence IBD progression. In animal models, high levels of taurocholic acid led to a 20% increase in sulfite-producing bacteria and worsened disease symptoms [24]. Moreover, the farnesoid X receptor (FXR), involved in bile acid regulation, has been identified as a potential therapeutic target for IBD, with FXR agonists showing protective effects in mouse models of chemically induced colitis [16,27].

Overall, the development of IBD post-gastrectomy is a multifactorial process involving complex interactions between genetic, environmental, and physiological factors. Understanding these mechanisms can help in developing targeted strategies to mitigate the risk and improve patient outcomes.

### 3.4. Inflammatory Implications of Microbial Shifts

The role of the microbiome in inflammation, particularly within the gastrointestinal context, is intricate and multifaceted. The gut microbiota, comprising approximately 100 trillion microbes, plays a crucial role in immune system regulation, nutrient absorption, and pathogen defense. Changes in the gut microbiome can profoundly impact these functions, leading to inflammation and related disorders. Bariatric surgeries, such as laparoscopic Roux-en-Y gastric bypass (LRYGB) and sleeve gastrectomy (SG), offer a unique perspective on how surgical interventions can alter the gut microbiota and influence inflammation (Table 2).

Post-bariatric surgery, significant changes in gut microbiota composition and function have been observed. One of the most critical divergences is the extent of the increase in *Proteobacteria* species, particularly *E. coli* and *K. pneumoniae*. Both species are notably increased after surgery, with a more pronounced increase following LRYGB compared to SG, confirming results from other studies [28]. The increase in *E. coli* may reflect the host and gut adaptation to maximize energy harvest under post-surgical, starvation-like conditions. Additionally, *A. muciniphila*, known to be negatively correlated with inflammation, increased in similar proportions in patients after both SG and LRYGB, corroborating findings from previous research [28]. The adherent-invasive *E. coli* (AIEC) pathotype is known for its pro-inflammatory effects, especially in patients with ileal Crohn’s disease (CD), where it is more prevalent compared to non-IBD controls. AIEC drives inflammation by altering the gut microbiota, leading to increased levels of bioactive lipopolysaccharides (LPS) and flagellin, reducing microbial diversity, and changing bacterial composition. This chronic inflammation is notably pronounced in individuals lacking the TLR5 receptor for flagellin. Additionally, AIEC exacerbates inflammation through mechanisms such as the elevated production of reactive oxygen species (ROS), the suppression of mucin gene expression, and increased levels of the chemotactic cytokine IL-8 [22].

Furthermore, pro-inflammatory anti-α-Gal antibodies, which can be induced by bacteria like *E. coli*, play a role in enhancing inflammation. The α-Gal antigen on certain bacteria triggers an immune response, leading to elevated levels of anti-α-Gal antibodies, particularly IgG. These antibodies bind to bacteria and activate immune responses, thereby exacerbating inflammation and contributing to the severity of inflammatory bowel disease (IBD) [22,29].

The number of metagenomics species pan-genome (MSP) negatively affected by bariatric surgery is lower. For instance, *F. prausnitzii*, a butyrate producer with anti-inflammatory properties, decreased by 6 months post-surgery in LRYGB patients, while SG had no significant effect on its presence in feces. This decrease in *F. prausnitzii* following LRYGB has been previously reported in two other studies. Similarly, *R. gnavus* and *R. torques*, known for producing trans-sialidase to degrade mucin and being associated with inflammatory bowel diseases and metabolic disorders, also decreased in LRYGB [28].

The gut microbial composition post-surgery includes an increased relative abundance of bacterial genera considered pathogenic and associated with colitis development. Notably, the class *Gammaproteobacteria* and its associated family *Enterobacteriaceae* show a significant increase, which parallels observations in human IBD patients [21]. In particular, *Escherichia* and *Shigella*, subdivisions of *Enterobacteriaceae*, are increased in both human patients and rodent models post-bariatric surgery, indicating a potential risk of increased colitis. Oral colonizers, such as *Veillonella* and *Streptococcus*, show a higher increase after LRYGB than SG, likely due to a reduced exposure to the acidic stomach environment [21,28].

The diversity of the gut microbiota, measured through species richness (alpha diversity), is significantly lower post-surgery. For instance, in the study by Imai et al., the observed OTUs (operational taxonomic units) and Shannon’s index showed significant reductions post-distal gastrectomy (DG) (*p* = 0.001 and *p* = 0.03, respectively). Fecal calprotectin, a marker of intestinal inflammation, was significantly higher in SGB2 patients compared to controls and positively correlated with the abundance of *Streptococcus* [4]. Conversely, beneficial bacteria like Ruminococcaceae, Barnesiella, and Anaerostipes, which negatively correlated with fecal calprotectin levels, showed decreased abundances [4,5,14].

The gut-associated lymphoid tissue (GALT), consisting of both isolated and aggregated lymphoid follicles, is a significant modulator of immune responses to microbial changes. In Crohn’s disease (CD), an abnormal Th1-mediated inflammatory response to commensal bacteria is observed [20]. The increased translocation of nonpathogenic *E. coli* and specific immune cell populations in GALT highlight the microbiome’s role in influencing immune responses and inflammation [20].

The functional capacity of the gut microbiota post-bariatric surgery shows enrichment in modules related to nutrient transport and metabolism. This shift is functionally relevant as it influences the host’s metabolic pathways, including energy harvest and fat metabolism. For example, beneficial bacteria such as *Lactobacillus* and *Bifidobacterium* were more abundant in certain surgical groups, while harmful bacteria like *Dialister* were more prevalent in others [25].

The microbiome’s impact on inflammation is profound, affecting both local gut health and systemic immune responses. Post-bariatric surgery changes illustrate the complex interplay between microbial composition, functional modules, and host immune responses. While some changes may offer metabolic benefits, the increase in pathogenic bacteria and reduced microbial diversity pose significant risks for inflammation and related disorders. Understanding these dynamics is crucial for developing targeted therapies to manage inflammatory conditions and improve gastrointestinal health.

Saliva can transfer to the digestive tract oral bacteria and relocate them, influencing gastrointestinal microbiota. *Fusobacterium nucleatum* and *Porphyromonas gingivalis* are some of the main bacteria that, after their translocation, are directly linked with IBD and colorectal cancer. These bacteria may affect homeostasis in the stomach and colon, contributing to inflammation and carcinogenesis. However, the mechanisms by which oral bacteria colonize the gastric mucosa and their role in health and disease remain unclear [3,30].

Oral bacteria like *Fusobacterium nucleatum* and *Porphyromonas gingivalis* can translocate to the gastrointestinal tract, contributing to diseases such as inflammatory bowel disease (IBD) and colorectal cancer [3]. In a studied group, 67.5% of patients experienced defecation problems, 40% had chronic constipation, and 30% had chronic diarrhea, highlighting the significant impact of microbial dysbiosis on gastrointestinal health.

Patients with gastrointestinal complaints had a higher relative abundance of *Enterobacteriaceae* (linked to inflammatory conditions like IBD, obesity, colorectal cancer, and celiac disease), *Parasutterella* (associated with IBS and chronic intestinal inflammation), and *Enterococcus* (opportunistic pathogens causing infections, particularly *E. faecalis* and *E. faecium*, related to serious complications and nosocomial infections) [31]. A higher number of *Sellimonas*, which may benefit gut health, was also observed [31].

The gut microbiota in patients with gastrointestinal complaints was related to increased concentrations of isobutyric acid in the feces. This branched fatty acid (BCFA), produced by fermentation of branched-chain amino acids by gut bacteria such as *Bacteroides* and *Clostridium*, can provide energy for colonocytes when butyric acid is insufficient. However, an increase in BCFAs may indicate heightened proteolytic fermentation, leading to harmful metabolites like ammonia, p-cresol, phenols, and hydrogen sulfide. These metabolites can disrupt colon epithelium, causing mucosal inflammation, affecting the intestinal nervous system and motility, and contributing to conditions such as inflammatory bowel diseases and colorectal neoplasms [31].

## 4. Discussion

Gastrectomy is the primary treatment for gastric cancer (GC) and significantly improves patient survival rates. However, postoperative complications, including inflammatory bowel disease (IBD), substantially impact recovery and currently lack effective treatment measures. Our study faces certain limitations that introduce potential biases, primarily due to the lack of sufficient data on patients who have undergone gastrectomy. The sample size of individuals studied is relatively small, making it difficult to draw broad, generalizable conclusions about the relationship between gastrectomy and subsequent gastrointestinal issues such as inflammatory bowel disease (IBD). While we have attempted to draw meaningful insights from the available data, the limited number of subjects reduces the statistical power and reliability of our findings.

Additionally, although the various types of gastrectomy procedures share similarities, the underlying pathologies and surgical techniques can differ significantly, complicating our ability to compare and standardize outcomes across the patient population. This heterogeneity in surgical methods and patient conditions adds complexity to interpreting the results, as the influence of these differences on gut microbiota alterations and the risk of developing IBD remains unclear. Therefore, while this study offers valuable insights, further research involving larger, more diverse patient cohorts is necessary to fully understand the impact of gastrectomy on long-term gut health. Many treatment and management strategies have been described in the literature.

Zheng et al. conducted a study on the efficacy of probiotic compounds (four strains: *Lactobacillus* plantarum MH-301, *L. rhamnosus* LGG-18, *L. acidophilus*, and *Bifidobacterium* animalis subsp. lactis LPL-RH) as auxiliary treatment measures post-gastrectomy. Clinical research results demonstrated that these probiotics significantly reduced postoperative inflammation, enhanced immunity, restored gut microbiota composition, and promoted recovery (this was a strong suggestion) [32]. In another clinical trial by Zheng et al., involving 100 gastric cancer patients, probiotics were administered to patients undergoing partial gastrectomy. The probiotics group showed a significant decrease in inflammation index (leukocytes), enhanced immunity (lymphocytes), and improved nutritional indices (albumin and total protein) compared to the placebo group. Fecal analysis revealed an increased relative abundance of *Bacteroides*, *Faecalibacterium*, and *Akkermansia*, and a decreased relative abundance of *Streptococcus* in the probiotics group [1]. Animal model results indicated that gastrectomy led to increased inflammation, impaired immunity, and disrupted gut microbiota. However, administering probiotics downregulated inflammatory and permeability signaling pathways, reduced pro-inflammatory factors, maintained the intestinal mucosal barrier and immune function, and restored gut microbiota homeostasis.

Park et al. highlighted additional studies supporting the benefits of probiotics post-gastrectomy. In a study by Cao et al. (2019), 100 patients were randomized to receive *Clostridium butyricum* or placebo for up to 21 days post-surgery. In the *C. butyricum* group, a depletion in the levels of leukocytes, neutrophils, IL-1β, IL-6, and TNF-α and higher levels of immunoglobulins, lymphocytes, albumin, and total protein were observed, compared to the placebo group. Butyric acid, the primary metabolite of *C. butyricum*, serves as a crucial nutrient for the regeneration and repair of intestinal epithelial cells, with its activity remaining unaffected by gastric and bile acids. Fecal analysis demonstrated increased levels of beneficial bacteria, including *Bacteroides*, *Faecalibacterium*, and *Gemmiger*, alongside reduced levels of *Streptococcus*, *Desulfovibrio*, and *Actinomyces* (this was a moderate suggestion) [6,10].

Triantafylidis et al. emphasized the importance of enhanced recovery after surgery (ERAS) and enteral nutrition (EN) pre-surgery. A study involving 200 GC patients compared postoperative outcomes between those receiving EN one week preoperatively (study group) and those starting EN postoperatively (control group). The study group showed significantly better immune responses and reduced inflammatory responses. Another study of 106 GC patients corroborated these findings, showing improved postoperative nutritional status and immune parameters in patients receiving preoperative nutritional support [14,33]. Deep neuromuscular blockade (NMB) with low intra-abdominal pressure is an essential component of enhanced recovery after surgery (ERAS) in gastrointestinal surgeries, particularly for its role in mitigating postoperative intestinal dysfunction. Deep NMB has been shown to improve surgical conditions in laparoscopic procedures by reducing the demand for CO_2_ insufflation. It is especially recommended for laparoscopic surgeries near the diaphragm, such as cholecystectomy or gastrectomy [25].

Diagnosing post-surgery IBD is challenging. Dumping syndrome and diarrhea from malabsorption or quick passage through the gastrointestinal tract can be observed with the same symptoms as IBD, such as diarrhea, discomfort, bloating, and atypical abdominal pain. The need for biomarkers for the diagnosis is crucial. Westernik et al. discussed the use of fecal biomarkers to measure intestinal inflammation post-surgery. Fecal calprotectin (fCP), calgranulin-C, and lactoferrin levels correlate with clinical disease activity scores and tissue inflammation in IBD patients. After Roux-en-Y gastric bypass (RYGB), median calprotectin levels were found to significantly increase, indicating heightened intestinal inflammation. These biomarkers help distinguish between IBD and other conditions, aiding in postoperative management [34]. Igwe et al. provided a mechanistic explanation for postoperative inflammation, suggesting a pro-inflammatory, pro-mitotic state within the colonic mucosa. This state may result from altered bile circulation and micronutrient deficiencies, such as magnesium. Aspirin’s anti-inflammatory and immunomodulating effects were highlighted, with long-term aspirin use associated with reduced epigenetic aging and lower odds of IBD [20]. Fecal microbial translocation is under investigation for its anti-inflammatory role. Yao et al. explored the effects of FMT in mitigating postoperative colitis. R-Y FMT reduced intestinal inflammation and improved nutrient absorption in mice, compared to B-I and B-II FMT groups. FMT with SCFA-producing bacteria downregulated the NLRP3 signaling pathway, inhibiting macrophage activation and reducing pro-inflammatory mediators like caspase-1 and IL-1β [15].

Probiotics and preoperative nutritional strategies show promise in enhancing postoperative recovery and managing inflammation in gastric cancer patients undergoing gastrectomy. Combined probiotics with different species of *Lactobacillus*, *B. cereus*, *E. faecalis*, *Bifidobacterium* or *Clostridium butyricum* have shown positive and encouraging results regarding maintaining a balance in the gut microbiome, increasing the percentage of inflammation-mediating bacteria, and reducing the percentage of bacteria related to IBD. The utilization of probiotics at high doses for a duration of 7 days, starting at the 3rd postoperative day, has proven to be the most beneficial [1]. Further research is needed to fully understand their potential and optimize treatment protocols.

We calculated the Grading of Recommendations Assessment, Development and Evaluation GRADE score for the publications included in our study based on key outcomes, including the incidence of inflammatory bowel disease (IBD) after bariatric surgery (BS), IBD after gastrectomy, and microbial shifts following gastric surgery. The quality of evidence for each outcome was assessed following the GRADE framework, considering factors such as study design, risk of bias, consistency, heterogeneity, relation to our question, and precision. This approach allowed us to provide a clear evaluation of the strength of the evidence supporting each outcome. Most of the articles utilized had moderate-quality evidence (13/34) and, according to the literature, there is strong evidence for the use of probiotics after gastrectomy. However, the correlation between gastrectomy for gastric cancer and IBD still remains unclear.

## 5. Conclusions

Gastrectomy significantly alters the gastrointestinal environment, leading to reduced gastric acid production and changes in the gut microbiota. These alterations are linked to an increased risk of inflammatory bowel disease (IBD) and other postoperative complications. The increased presence of oral and aerotolerant bacteria, along with changes in bile acid composition, contribute to systemic inflammation and intestinal dysbiosis. Probiotic interventions and enhanced recovery protocols show promise in mitigating these effects, improving postoperative outcomes, and restoring gut microbiota balance. However, further research is needed to better understand the optimal strains, dosage, and timing of probiotic use in post-gastrectomy patients. Large-scale clinical trials are crucial to assess the long-term efficacy of these interventions. Additionally, investigating how different probiotic formulations interact with bile acid metabolism may provide insights for more effective treatments. Further research is essential to fully understand the long-term implications of gastrectomy on gut health and to develop targeted therapies for optimal patient care.

## Figures and Tables

**Table 1:** miRNAs in gastric cancer and inflammatory bowel disease.

Mi-RNA	Role in Gastric Cancer and IBD
miR-29	Regulates immune response, affects IL-23 levels in dendritic cells.
miR-223	Modulates dendritic cells and macrophages, reduces inflammation.
miR-146b	Controls macrophage polarization and inflammation.
miR-150	Regulates immune cell development and intestinal barrier integrity.
miR-155	Enhances T-cell responses and NK cell function, contributing to inflammation.
miR-24	Influences T-cell development and apoptosis, impacting inflammation.
miR-29b-1-5p	Affects gastric cancer progression and intestinal epithelial cell apoptosis.
miR-30c	Regulates autophagy, Th17 cell differentiation, and macrophage behavior under hypoxic conditions.
miR-106b	Linked to disease severity in Crohn’s disease and UC, affecting cell invasion and inflammation.
miR-141-3p	Prevents normal fibroblast transformation into cancer-associated fibroblasts, plays a role in immune responses.
miR-199a-5p	Promotes cancer progression and contributes to inflammation and stress.

**Table 2:** Role of bacteria in inflammation.

Bacteria	Role in Inflammation
*Proteobacteria* (*E. coli*, *K. pneumoniae*)	Significantly increased post-bariatric surgery (especially after LRYGB). *E. coli* may adapt to maximize energy harvest post-surgery and is linked to inflammation.
*Akkermansia muciniphila*	Negatively correlated with inflammation. Increased post-surgery in both SG and LRYGB, indicating potential anti-inflammatory effects.
*Faecalibacterium prausnitzii*	Butyrate producer with anti-inflammatory properties. Decreased 6 months post-LRYGB but not significantly affected by SG, leading to a reduction in its protective effects.
*Ruminococcus gnavus* and *Ruminococcus torques*	Associated with inflammatory bowel diseases (IBD) and metabolic disorders. Decreased post-LRYGB, potentially reducing inflammation related to these species.
*Enterobacteriaceae* (*Escherichia*, *Shigella*)	Increased post-bariatric surgery. Associated with colitis and observed in both human patients and rodent models, indicating a risk for inflammatory conditions like IBD.
*Veillonella* and *Streptococcus*	Increased post-LRYGB, possibly due to reduced exposure to acidic stomach environments. Streptococcus correlates with higher fecal calprotectin levels, a marker of inflammation.
*Ruminococcaceae*, *Barnesiella*, and *Anaerostipes*	Beneficial bacteria negatively correlated with fecal calprotectin levels. Their decreased abundance post-surgery may contribute to increased inflammation.
*Fusobacterium nucleatum*	Oral bacteria that can translocate to the gastrointestinal tract, contributing to diseases like inflammatory bowel disease (IBD) and colorectal cancer.
*Porphyromonas gingivalis*	Oral bacteria that can translocate to the gastrointestinal tract, associated with inflammation and diseases such as IBD and colorectal cancer.
*Parasutterella*	Linked to chronic intestinal inflammation and irritable bowel syndrome (IBS). Increased in patients with gastrointestinal complaints.
*Enterococcus* (*E. faecalis*, *E. faecium*)	Opportunistic pathogens linked to infections and serious complications. Increased in patients with gastrointestinal complaints and associated with inflammatory conditions.
*Sellimonas*	Potentially beneficial to gut health. Observed in higher numbers in patients with gastrointestinal complaints, but specific anti-inflammatory effects are not clearly defined.
*Bacteroides* and *Clostridium*	Associated with the production of branched-chain fatty acids (BCFAs) through proteolytic fermentation. Increased BCFAs may lead to harmful metabolites contributing to inflammation.
*Lactobacillus* and *Bifidobacterium*	Beneficial bacteria associated with improved gut health and reduced inflammation. Increased in certain post-bariatric surgery groups.
*Dialister*	Harmful bacteria that were more prevalent in some post-surgical groups, potentially contributing to inflammation.
